# Functional Disorder at the Neural Interface: How Disordered Nanostructures Promote Proper Growth and Differentiation in In Vitro Neural Cultures

**DOI:** 10.1002/advs.202600024

**Published:** 2026-04-13

**Authors:** F. Maita, E. Palmieri, I. Lucarini, L. Montaina, L. Maiolo

**Affiliations:** ^1^ Istituto per la Microelettronica e Microsistemi Consiglio Nazionale delle Ricerche Rome Italy

**Keywords:** disordered nanostructures, fractal nanotopography, functional disorder, in vitro neural culture, neural and glial interfaces

## Abstract

The performance and reliability of neural interfaces critically depend on the ability to engineer electrode‐tissue interactions across multiple length scales. In this review, we introduce functional disorder as a new unifying design paradigm for manufacturing inorganic nanostructured biointerfaces. We introduce the term functional disorder to denote a non‐periodic, spatially heterogeneous, multiscale nanotopography whose irregular geometry is functionally relevant at the cell‐electrode interface. We focus on how tailored nanotopography can be exploited to modulate key interfacial properties relevant to in vitro neural platforms, including effective impedance reduction, enhanced electrical coupling, and improved signal recording from both neuronal and glial populations. Beyond electrical performance, functionally disordered nano‐architectures provide complex topographical and mechanical cues that influence cell adhesion, morphology, and differentiation, enabling more stable and physiologically relevant interfaces. By critically analysing fabrication strategies and structure‐property relationships without restricting the discussion to specific material systems, this review establishes general principles linking disorder, interfacial functionality, and biological response. Overall, we propose functional disorder as a rational and scalable framework to guide the design of next‐generation in vitro neural interfaces with improved performance, robustness, and biological integration.

## Graphical abstract

This work provides a practical guide for neuroengineers to design advanced neural interfaces, embracing and tailoring the concept of *functional disorder*. By bridging 2D and 3D in vitro models, this work highlights how non‐periodic, spatially heterogeneous, multiscale nanotopography can enable more physiologically relevant platforms for studying neural growth, connectivity, and activity.

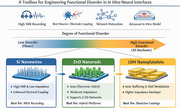

## 1 Introduction

For decades to design and manufacture bio‐interfaces, materials engineering has pursued order as the ultimate expression of control: atomically flat films, periodic nanopatterns, and highly reproducible surface geometries were believed to offer predictable biological outcomes [1, 2, 3]. Yet, the biological world is inherently irregular. The extracellular matrix (ECM) is not a crystal lattice: it is a dynamic, heterogeneous network of fibres, pores, and chemical gradients. Its “imperfection” is precisely what confers resilience, adaptability, and functionality. In contrast, perfect order often reduces the complexity of biophysical signalling, leading to oversimplified or unstable biological responses in vivo. This discrepancy has prompted a conceptual shift: from precise order to functional disorder. In this Review, we use the term functional disorder to denote a non‐periodic, spatially heterogeneous, multiscale nanotopography whose irregular geometry is functionally relevant at the cell‐electrode interface. Specifically, this morphology can simultaneously increase anchoring opportunities for cells, modulate protein and extracellular‐matrix organization together with integrin‐mediated mechanotransductive signalling, expand the surface available for localized biofunctionalization, and‐ when porosity is present‐ facilitate ionic and soluble‐species exchange. The notion of functional disorder can be further extended to describe stochastically organized, fractal or pseudo‐fractal architectures that emulate the statistical self‐similarity observed in biological systems. In nature, tissues such as pulmonary bronchioles, neuronal dendritic trees, or vascular networks display hierarchical branching patterns that are not perfectly self‐similar but follow statistical scaling laws over multiple length scales [4]. These stochastic fractal geometries maximize surface‐to‐volume ratio, transport efficiency, and mechanical adaptability while maintaining structural robustness, aspect that ordered artificial architectures rarely achieve [5]. In this emerging view, irregularity is no longer a manufacturing artefact but a design parameter that can be engineered and quantified. Disordered nanostructures, characterized by statistical distributions of roughness, curvature, porosity, and atomic defects, can reproduce the stochastic cues that cells experience in natural tissues. Such bioinspired randomness has been shown to promote more robust adhesion, spontaneous differentiation, and enhanced tissue integration compared to perfectly periodic surfaces [6, 7, 8, 9, 10, 11]. Indeed, biological tissues exhibit hierarchical stochastic architectures that range from the fibrillar disorder of collagen to the irregular mineral topography of bone. These multiscale heterogeneities distribute mechanical stress, enhance biochemical signalling and provide redundancy against failure. Such properties cannot be reproduced by ordered arrays of nanofeatures.

In contrast, highly periodic nanotopographies often induce synchronized yet limited cellular responses: cytoskeletal alignment, constrained migration, and partial differentiation [12, 13]. Living systems, however, depend on heterogeneity and noise to maintain plasticity and homeostasis. Functional disorder thus becomes a way to reintroduce “biological realism” into engineered materials.

**FIGURE 1:**
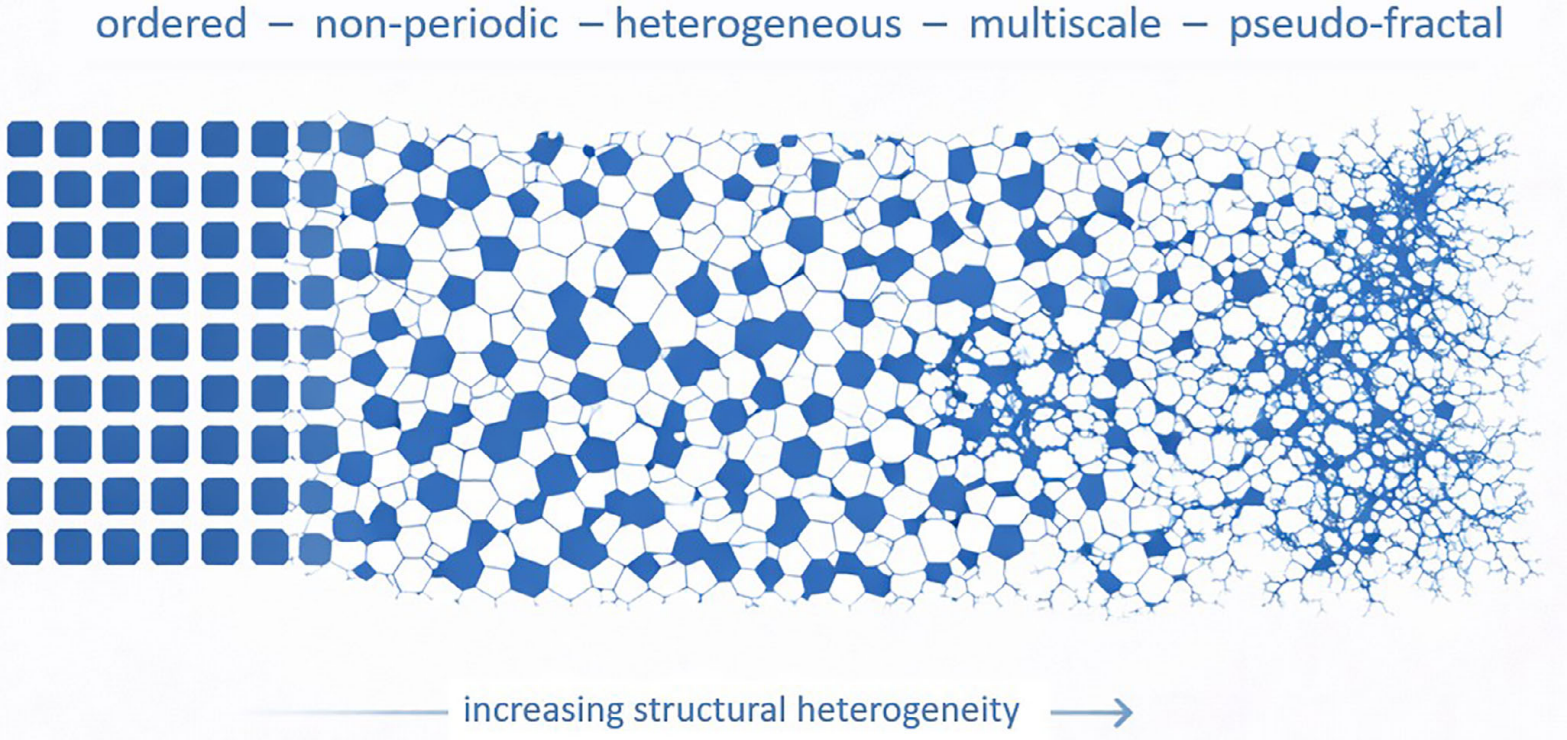
Top‐view schematic representation illustrating increasing non‐periodicity, multiscale roughness and hierarchical heterogeneity from ordered nanopatterned arrays to pseudo‐fractal nanostructures. The schematic is intended as a conceptual map of interfacial complexity, not as a one‐dimensional definition of disorder.

At the material level, disordered inorganic nanostructures exhibiting fractal‐like topographies or roughness spectra with power‐law behaviour replicate this principle. Their non‐integer dimensionality and scale‐invariant roughness promote multi‐scale interactions with proteins, ions, and cells, effectively coupling nano‐, micro‐, and mesoscopic phenomena [14, 15]. Such pseudo‐fractal disorder thus represents not random chaos but an optimized complexity, in which structural irregularity becomes a route to enhance biochemical exchange, charge transport, and cellular communication across the bio‐inorganic interface [16]. In this sense, functional disorder can be interpreted as the material analogue of biological self‐organization—a stochastic order that serves function rather than form (see Figure 1).

**FIGURE 2:**
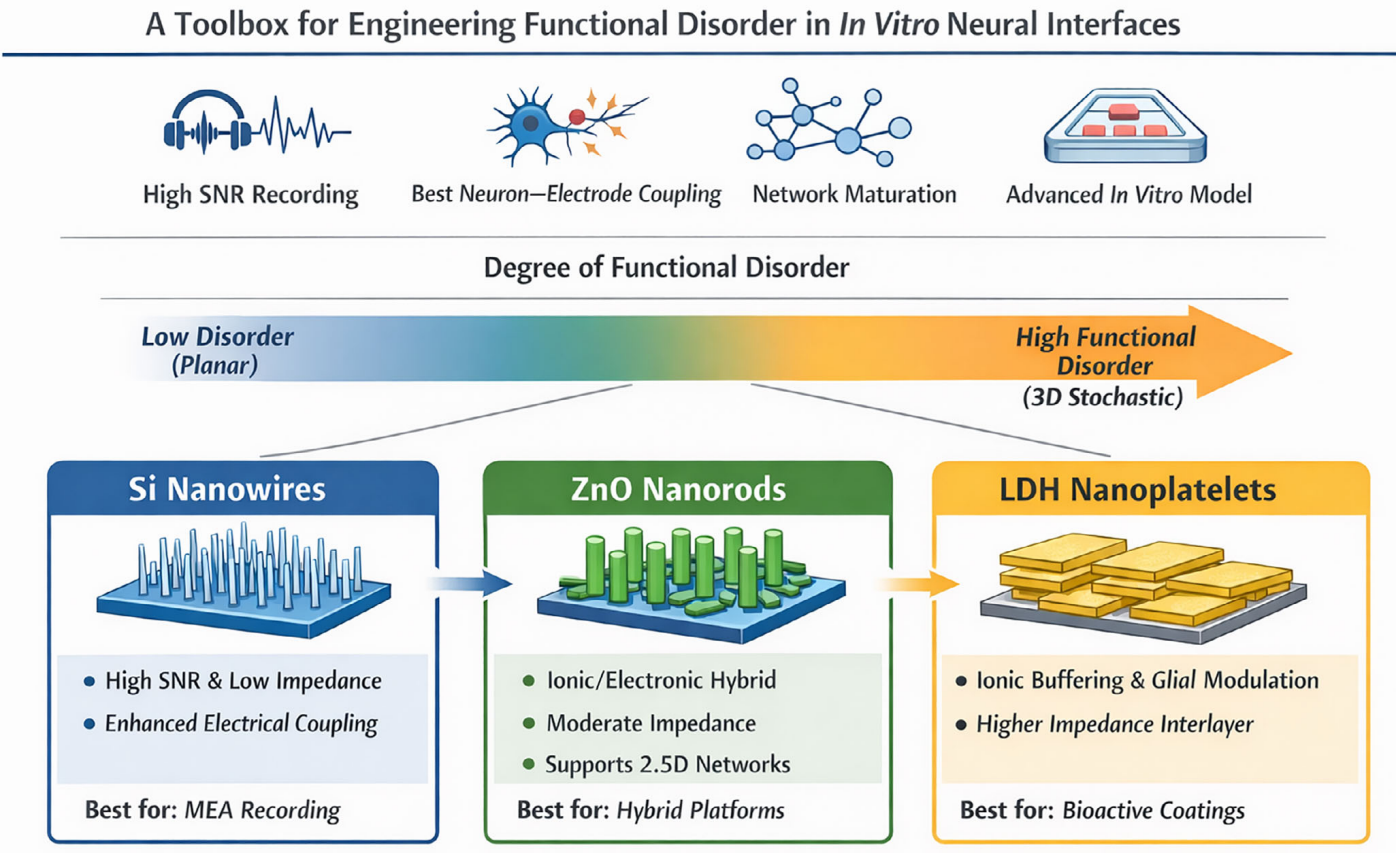
Design‐oriented toolbox illustrating how different classes of disordered inorganic nanostructures can be selected based on targeted electrophysiological performance in advanced in vitro neural models. Rather than representing a single optimal solution, the toolbox highlights complementary regimes of functional disorder enabling tailored bioelectronic interfaces.

In neuroscience, the concept of functional disorder is particularly critical for in vitro systems, where the formation of functional neuronal networks is indispensable to establish bio‐interfaces that allow natural cell behaviour and yield time‐resolved insights into physiological and pathological mechanisms. Disordered nanotopographies offer distinct advantages when applied to neural and glial interfaces [17], where the integration between soft biological tissue and rigid electronic or inorganic substrates remains a critical challenge [18]. In contrast to planar or periodically patterned electrodes, stochastically structured surfaces provide a topographical and mechanical heterogeneity that better mimics the microarchitecture of the neural extracellular matrix [19]. At the cellular level, these irregular 2D and 3D landscapes promote intimate membrane conformability, reduce mechanical mismatch, and enhance neurite anchoring and outgrowth. Astrocytes and microglia, which are highly sensitive to surface roughness and chemistry, exhibit attenuated activation on such disordered nanostructures, leading to reduced inflammatory encapsulation and improved chronic stability in vivo. From a functional point of view, the integration of disordered and conductive nanoelectrodes in the bio‐interface significantly lowers electrode impedance, enhances charge‐transfer efficiency, and improves signal‐to‐noise ratios during electrophysiological recording or stimulation. When extended into 3D porous scaffolds, the same architecture facilitates nutrient transport, oxygen diffusion and integration with microfluidic systems, enabling hybrid neural organoids or brain‐on‐chip models with realistic biophysical microenvironments. Disordered inorganic nanomaterials, such as Silicon Nanowire forests (SiNWs), metal oxide nanograss, nanorods, nanowires (nano‐MeOx), nanofibers, porous silicon or bioactive glasses, exemplify this principle at the material level [7, 11, 14, 20, 21, 22]. Their stochastic architecture maximizes surface area, enhances wettability, and facilitates the formation of complex protein coronas that mediate cell recognition. Moreover, atomic‐scale disorder (e.g., oxygen vacancies or mixed oxidation states) tunes the surface charge and redox reactivity, further influencing cell adhesion and biocompatibility. To guide neuroengineers in selecting and engineering disordered nanostructures based on targeted electrophysiological performance, Figure 2 summarizes functional disorder as a practical toolbox rather than a single optimal solution.

From a functional perspective, disordered architectures provide synergistic mechanical and electrical advantages. High‐surface‐area electrodes based on nanoporous gold [23], “cauliflower‐like” TiN [24], or nanostructured carbons [25] exhibit drastically reduced impedance, without the need of using conductive polymers (CPs), particularly mixed ionic‐electronic conductors (MIECs) such as PEDOT and polypyrrole, that have been extensively employed to reduce electrode impedance [26, 27]. Simultaneously, the interconnected porosity of anodic aluminium oxide (AAO), disordered sol‐gel oxides, or black silicon promotes fluid transport and nutrient exchange, a crucial aspect for the design of in vivo‐like microenvironments and organ‐on‐chip systems [28]. Hence, the same stochastic geometry that benefits biological adhesion also enables multimodal integration, coupling biological, electrical and fluidic functions within a single material platform.

This review is conceived as a practical toolbox for neuroengineers, providing design rules and comparative metrics to guide the selection and engineering of disordered inorganic nanostructures for high‐performance in vitro neural interfaces. In particular, we explore how functional disorder, spanning from topographical irregularity to atomic‐scale heterogeneity, can be deliberately engineered in inorganic materials to properly interact with the biological world and, in particular, with neural and glial cell networks. We systematically discuss (i) quantitative descriptors of disorder beyond conventional RMS roughness, (ii) correlations between nanoscale topology and cellular mechanotransduction, (iii) the methods to synthesize and integrate disordered nanomaterials in platform for interrogating the brain, (iv) influence of disorder on cell growth and differentiation to obtain in vivo like cell cultures with behaviour comparable with in vivo models (v) influence of these structures on impedance reduction. Accordingly, this review focuses on disordered inorganic nanostructures, whose predominantly electronic conduction and geometry‐driven cell‐electrode coupling provide specific recording fidelity, interfacial stability, and reproducibility compared to ionically conducting polymer‐based systems, whose electrochemical response is governed by fundamentally different charge transport mechanisms.

## 2 Operational Definition and Descriptors of Functional Disorder

Because functional disorder is a broad interfacial concept, no single metric can exhaustively capture it. In the following sections, we therefore discuss complementary descriptors, including structural features like non‐periodicity, multiscale roughness, porosity/interconnectivity, and local chemical heterogeneity. All these features provide a specific interfacial function that can be crucial for the proper growth and differentiation of a cell culture (see Table 1). Within this framework, fractal dimension is introduced as one useful descriptor of roughness and space‐filling complexity across scales [29, 30], alongside the biological and electrochemical functions that emerge from these structural features.

**TABLE 1:** Structural features, operational descriptors and functional outcomes of functional disorder at the neural interface.

Structural feature	Possible descriptor	Interfacial function
Non‐periodicity	Nearest‐neighbour spacing	Multiple cell anchoring opportunities
Multiscale roughness	Fractal dimension	Protein/ECM organization
Porosity/Interconnectivity	Pore‐size distribution/Number of interconnections among cells	Ionic exchange reservoir/Signal exchange through the network
Atomic‐scale heterogeneity	Oxidation‐state distribution	Surface charge, different types of functionalization
Quasi‐3D architecture	Effective nanostructure stiffness	Reduced mechanical mismatch at cell/structure interface

## 3 Fractal Descriptors of Multiscale Roughness in Functionally Disordered Nanostructures

Among the descriptors of functional disorder, fractal dimension is particularly useful because it provides a scale‐free description of multiscale roughness and space‐filling complexity, even if it cannot be taken as the sole metric of surface irregularity [31]. The non‐periodicity, multiscale roughness, and high level of interconnectivity of the neural tissue finely match with complex disordered material interfaces that resemble the intrinsic geometrical nature of these organic structures. Indeed, from the biological standpoint, non‐periodic multiscale substrates provide a distribution of local curvatures, feature sizes, and pore configurations that can better accommodate the exploratory behaviour of neurons and glia than strictly periodic arrays. In particular, the morphological complexity of neuronal arbors can be quantitatively characterized by their fractal dimension (D), a parameter capturing how dendritic structures fill space across scales. In neuronal systems, D typically ranges between 1 and 3, although empirical studies indicate that it seldom exceeds 2. For instance, CA1 pyramidal neurons in the rat hippocampus exhibit relatively low median arbor fractal dimensions (D_A_), with values of 1.41 for basal and 1.42 for apical arbors [32]. Likewise, healthy retinal bipolar neurons display a mean covering fractal dimension of approximately 1.47 ± 0.01. Importantly, D is not a fixed property but reflects the balance between fine and coarse dendritic features, serving as an indicator of morphological optimization that mediates the trade‐off between potential synaptic connectivity and the metabolic and structural costs of sustaining complex arborization. Alterations in dendritic ramification significantly modulate D: computational models show that changes in dendritic weave (θ) and forking (ϕ) angles produce systematic variations in D_A_. While individual dendritic branches exhibit near‐linear geometries (branch fractal dimension D_B_ ≈ 1), increasing their tortuosity raises D_A_, whereas straightening reduces it. Developmentally, neuronal maturation in culture correlates with size‐dependent shifts in D: smaller, immature retinal bipolar neurons (R < 10 µm) present higher mean values (1.53) than larger, mature cells (1.44 for R > 20 µm) [32]. This trend suggests that immature neurons initially favour dense, local connectivity (high D), subsequently lowering D as arbors expand during maturation. Deviations from this natural equilibrium, such as those induced by artificial substrates or pathological remodelling of dendritic geometry, may thus displace D from its optimized physiological range.

In this respect, functionally disordered nanostructures offer distributions of feature size, local curvature and porosity that partially overlap the multiscale occupancy of neural and glial processes, thereby providing a more permissive environment for adhesion, outgrowth and maturation. Tuning nanostructures' length, width and level of intergrowth, it is possible to provide a unique environment for neural cell cultures offering specific nanotopography and pore structure to trap nutrients and favour ion exchanges [33]. We report below the value of D for inorganic disordered nanostructures (see Table 2) used as valuable cell substrates (silicon nanowires, ZnO nanorods, layered double hydroxide nanoplatelets) in neural and glial cell cultures. In this respect, we must point out that polylysine or other adhesion layers were not implemented and cell cultures expressed in vivo‐like behaviour in cultures inferior of 10 days. Interestingly, the range D of these nanostructures is quite narrow and by modifying the length of the nanorods or nanowires, the results progressively worsen. Rather than numerically matching the exact fractal dimension of cells, these structures offer a consistent general architecture that responds to different cell types (e.g. neurons and astrocytes), allowing the emergence of remarkable physiological features in the proposed cell cultures.

**TABLE 2:** Fractal dimension calculated for the main disordered nanostructures treated in the review.

Type of nanostructure	Fractal Dimension	Standard Deviation
Silicon Nanowires	1.85	0.07
MgFe LDH	1.86	0.05
ZnO Nanowires	1.73	0.05
ZnO Nanorods	1.88	0.04

The fractal dimension of disordered nanostructures, such as forests of nanowires or nanorods, was determined from SEM images using the box‐counting method. The analysis began by converting the grayscale SEM micrographs into binary images to distinguish the nanostructures from the background. To ensure objective thresholding, Otsu's algorithm was applied, which automatically selects the optimal threshold by minimizing the intra‐class variance between foreground and background pixel intensities [34]. Once the binary image was obtained, the box‐counting algorithm was implemented: the image was covered with grids of boxes of different sizes, and for each grid size, the number of boxes containing part of the structure was counted. The relationship between the number of occupied boxes and the box size follows a power law, and the slope of the corresponding log‐log plot provides the fractal dimension (D). This procedure quantifies the degree of surface complexity and self‐similarity of nanostructure morphology. The obtained values of D, ranging from 1.73 to 1.88, indicate a highly irregular and complex topology typical of disordered nanostructured films.

## 4 Functional Disorder on Mechanical Properties of Nanostructures at the Interface

**FIGURE 3:**
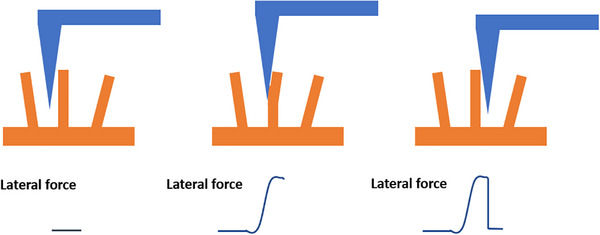
A simple sketch explaining the bending of a nanowire for determining the nanomaterial stiffness.

In particular, the lower stiffness of these distributed quasi 3D nanostructures with respect to the bulky materials offers a softer surface for cell adherence and process growth. Indeed, even for inorganic materials like silicon or zinc oxide, where the stiffness is very far from the tissue material (50–100 GPa vs 1–10 kPa), the nanostructures present significant lower values (around 100 MPa to 1 GPa) depending by the height of the nanostructures (from 100 nm to 1um) and the final nanowire diameter (<100 nm) as reported in AFM measurements (see figure 3). The effective mechanical response of nanowire carpets can be modelled using classical Euler‐Bernoulli beam theory, as in AFM bending experiments on individual nanowires [35, 36, 37], where the nanowire stiffness $k=\frac{3EI}{L^{3}}$ is extracted directly from AFM force‐deflection curves. When the nanowires are not cylindrical but linearly tapered (conical frustums), with a base radius R and tip radius r_t_, the local second moment of area becomes $I\;\;\left(z\right)=\frac{\pi }{4}\;{\left[R-\frac{(R-r_{t})z}{L}\right]}^{4}$. Using Castigliano's theorem for a cantilever with a tip load, the effective bending stiffness is $k_{cone}=\frac{3EI_{eff}}{L^{3}}$, where $I_{eff}=\pi R^{3}r_{t}/4$. Hence, $k_{cone}=k_{cyl}\;\left(\frac{r_{t}}{R}\right)$, and the effective elastic modulus of the nanowire carpet scales accordingly as $E_{eff}^{cone}=E_{eff}^{cyl}\;\;\left(\frac{r_{t}}{R}\right)$. This simple relation shows that conical nanowires further soften the interface by a factor equal to the tip‐to‐base radius ratio. This is one concrete example of why the disorder is functional: the same non‐periodic quasi‐3D architecture that increases interfacial area also lowers the effective stiffness perceived by cells and supports process extension.

## 5 The Role of Disordered Nanostructures on Integrins

Integrins are heterodimeric transmembrane proteins that function as the primary molecular link between the extracellular milieu and the intracellular cytoskeleton, orchestrating adhesion dynamics, migration and outgrowth processes in both neurons and glial cells. Integrins provide a molecular “search engine” through which neural and glial cells interrogate disordered nanosurfaces, converting stochastic nanoscale cues into organized adhesion, traction and ultimately physiologic outgrowth patterns. Historically, the first mechanistic hints emerged when β1‐integrin dynamics were directly probed in migrating growth cones on laminin, revealing tight coupling between integrin engagement and the actin cytoskeleton, which serves as an early blueprint for how neurons translate adhesion into forward advance [38]. This concept matured into the modern view of integrin‐dependent point contacts as transient, motile adhesion units: increasing integrin activation can rescue otherwise diminished neurite extension on ECM ligands [39], while coordinated Rac1/RhoA signalling stabilizes point contacts and protrusions during neurite outgrowth [40, 41]. Disordered nanotopographies amplify these mechanisms because they present a statistical distribution of curvature and ligand/adsorbate spacing that promotes integrin nanoclustering and a high‐turnover adhesion regime (many small, dynamic adhesions rather than a few over‐mature, mechanically “locking” focal adhesions) consistent with the broader nanotopography literature linking altered integrin organization and focal‐adhesion architecture to downstream cytoskeletal mechanics [42, 43, 44]. In advanced neuron‐glia cultures, this matters as much for astrocytes as for neurons: astrocytes can dominate the interface by spreading and sealing electrodes, yet nanostructured coatings such as nanoporous gold reduce astrocytic surface coverage and adhesion strength through focal‐adhesion‐dependent effects [45], while preserving neuronal coverage and enhancing electrophysiological recording performance [46, 47]. More broadly, nanostructured electrode platforms have been shown to promote network maturation and connectivity alongside improved functional readouts [48], supporting the idea that functional disorder is not merely permissive but actively instructive and enabling integrin‐mediated exploration that yields more physiologic neurite growth while curbing astrocyte‐driven interfacial encapsulation.

## 6 Disordered Nanostructures: Manufacturing Strategies

**FIGURE 4:**
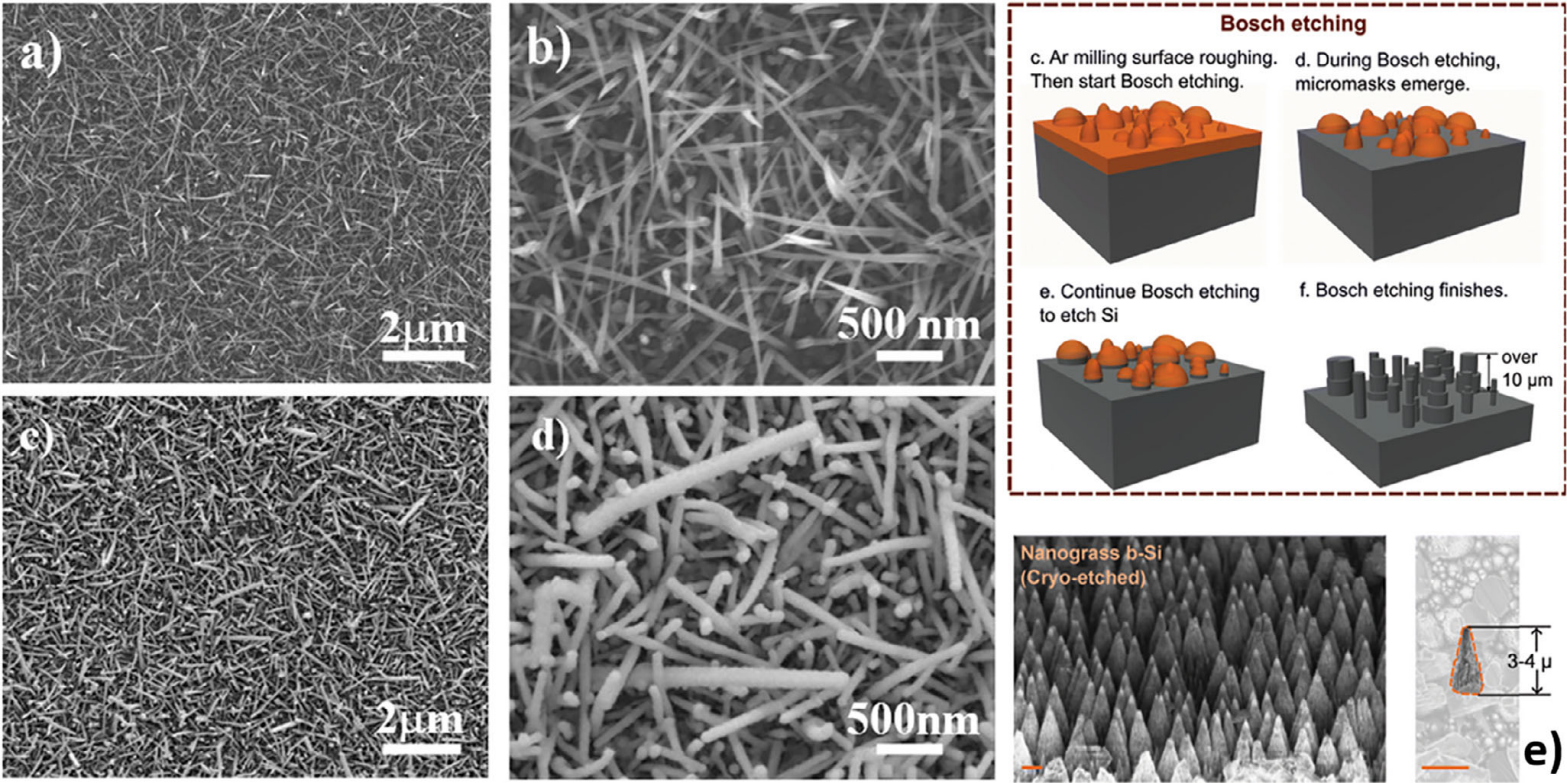
Top view SEM images of the as‐grown silicon nanowire forests (a,b) and after (c,d) Au coverage, courtesy of [58]. Reproduced with permission [58]. Copyright 2015, John Wiley & Sons. Bosch etching method for the fabrication of silicon nanograss e), courtesy of [59]. Adapted with permission [59]. Copyright 2025, RCS.

The fabrication of disordered inorganic nanostructures, such as silicon nanowires [49, 50, 51], iron oxide nanowires, zinc oxide nanorods [52, 53, 54] and layered double hydroxide (LDH) nanoplatelets [55, 56, 57], relies on a diverse portfolio of top‐down and bottom‐up techniques that enable stochastic, high‐surface‐area architectures across rigid and flexible platforms. Top‐down approaches such as deep reactive ion etching (DRIE), also called Bosch etching and metal‐assisted chemical etching (MACE) have proven exceptionally effective for generating “nanograss” silicon carpets with intrinsic randomness in height, density, and orientation (see figure 4e). RIE, in particular, is attractive for bioelectronic and flexible‐device applications because it operates at temperatures below 100°C and can be applied directly to polymeric substrates coated with thin Si layers, enabling mechanically compliant nanostructured interfaces. By contrast, classical bottom‐up VLS growth of silicon nanowires offers superior crystallinity but requires temperatures exceeding 400–900°C, limiting its integration on soft or plastic substrates. Plasma‐enhanced CVD represents a compromise, allowing nanowire nucleation at 250–450°C, marginally compatible with high‐temperature polymers such as polyimide (see figure 4a–d).

**FIGURE 5:**
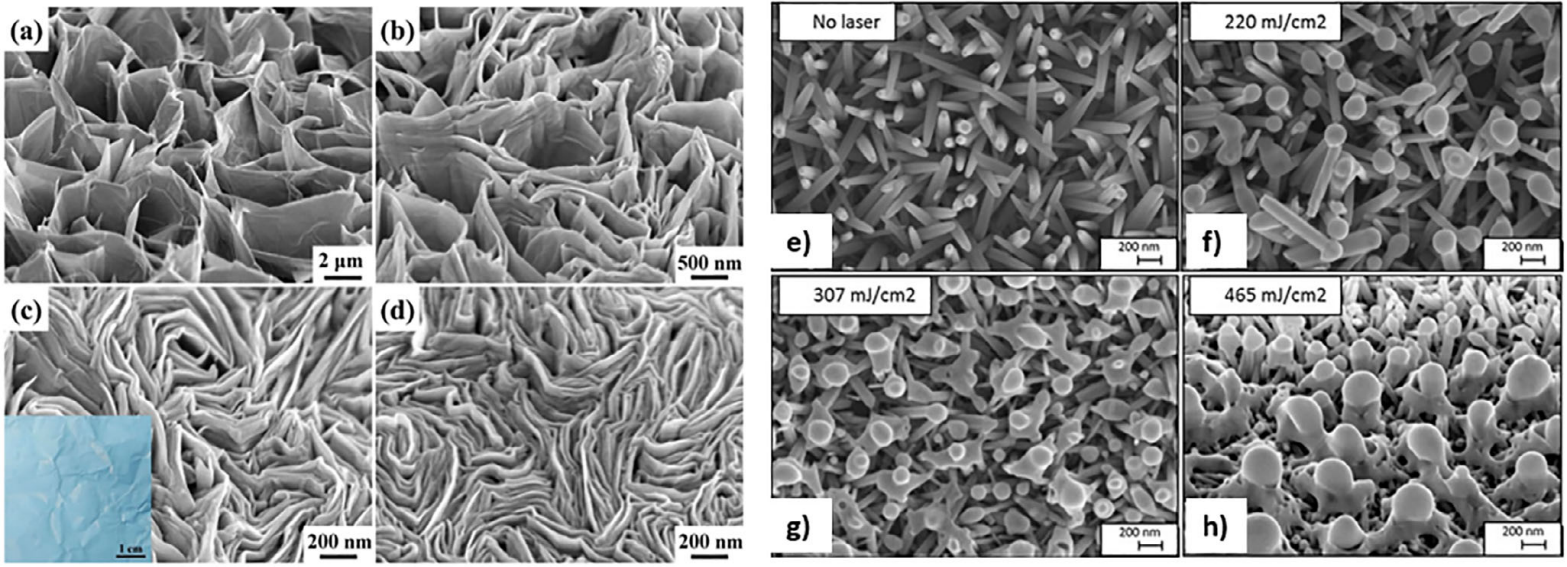
The tilt‐view FESEM images of ZnO nanowalls grown on the Al foil at different concentrations of ZnO precursors. The concentrations of ZnO precursors are (a) 10, (b) 25, (c) 50, and (d) 75 mm, respectively. Reproduced with permission [69]. Copyright 2022, MDPI. Courtesy of [69]. SEM images for disordered ZnO nanorods grown on Si oxide (e) and irradiated at (f) 220, (g) 307 and (h) 465 mJ/cm^2^ courtesy of [70]. Reproduced with permission [70]. Copyright 2018, Elsevier.

For metal‐oxide nanostructures, solution‐based bottom‐up methods provide a uniquely advantageous combination of morphological disorder, chemical tunability, and low‐temperature processing. For example, hydrothermal synthesis (HS) of ZnO nanorods proceeds at 70–95°C and yields dense, vertically oriented but intrinsically non‐uniform forests whose diameter, length, and aspect ratio vary stochastically across the surface. Such conditions naturally produce fractal‐like disorder while remaining fully compatible with plastic substrates, including PET, PI, PDMS, and paper. A simpler method with respect to the HS synthesis is called chemical bath deposition (CBD), where the reactions occur at atmospheric pressure, starting from a seed layer of ZnO nanoparticles or aluminium (see figure 5a–d). Additional low temperature deposition strategies (RT‐100°C) are related to microwave‐assisted hydrothermal synthesis [60, 61]. and sonochemical growth [62, 63]. where, through rapid heating or ultrasonic cavitation, it is possible to generate highly disordered nanostructures. Sol‐gel deposition and subsequent annealing (150–300°C) enable broader chemical versatility but impose tighter constraints on substrate selection. Conversely, physical vapor deposition techniques such as glancing‐angle deposition (GLAD) generate columnar, quasi‐fractal nanostructures due to self‐shadowing effects [64, 65]. These architectures exhibit tunable disorder and can be deposited at room temperature, offering a highly scalable platform for flexible electronics. Another way to change the nanotopography of the disordered structure is to use laser annealing in post‐processing [66]. By changing the exposure time, the beam energy density and the specific environment of irradiation (vacuum or controlled atmosphere) it is possible to modify the surface roughness and the shape of the nanostructures from sharp to smooth surfaces (see figure 5e,f). By prolonging the treatment or using higher energy, the nanostructures are flattened or ablated [67, 68].

LDH‐based nanoplatelets and nanoflakes, relevant for catalytic, sensing and bio‐interfacing applications, are readily synthesized via coprecipitation or electrodeposition at room temperature. These methods provide fine control over lateral dimensions, stacking order, and chemical composition while ensuring complete compatibility with polymeric or flexible substrates coated with conductive layers such as Au, ITO, or graphene. Hydrothermal crystallization of LDH at 80–180°C enables thicker, hierarchical morphologies with pseudo‐fractal features, though with more limited substrate compatibility. Together, these fabrication strategies highlight a recurring principle: functional disorder naturally emerges from growth conditions that relax spatial constraints, such as solution chemistry, ion transport, self‐shadowing or plasma‐induced stochastic erosion, rather than from deterministic lithographic control. By leveraging methods that operate in the low‐temperature regime (<100°C) and that inherently promote morphological heterogeneity, it becomes possible to engineer disordered nanostructured interfaces on soft, flexible and biocompatible substrates. Such architectures serve as ideal platforms for neural and glial interfacing, where mechanical compliance, high surface area and stochastic topography are critical for achieving intimate, long‐term coupling with living tissue.

## 7 Why Functional Disorder Emerges from Growth Kinetics

The emergence of functional disorder in inorganic nanostructures is not accidental but reflects the intrinsic interplay between growth kinetics, mass transport and local energetic landscapes. In top‐down processes such as RIE and MACE, stochastic fluctuations originate from competitive etching rates, micro‐masking phenomena and anisotropic ion‐surface interactions, resulting in nanoscale height and diameter variations that cannot be reproduced by deterministic lithography. Similarly, during bottom‐up solution‐based growth, such as hydrothermal synthesis of ZnO nanorods or coprecipitation of LDH nanoplatelets, the nucleation density, facet‐selective growth rates and diffusion‐limited aggregation processes produce morphological heterogeneity that naturally spans several length scales. These systems exhibit dynamic competition between growth fronts, local supersaturation gradients and defect‐mediated crystallization, leading to architectures that frequently display pseudo‐fractal signatures and statistical self‐similarity. Rather than degrading material performance, such disorder enhances surface‐to‐volume ratio, multiphase reactivity and local curvature distributions, giving rise to interfacial behaviors that are more compatible with the stochastic microenvironment of biological tissues [71]. Functional disorder therefore emerges as a direct consequence of non‐equilibrium growth conditions, where small perturbations, such as thermal fluctuations, precursor depletion, and ion diffusion anisotropy, are amplified into mesoscale patterns that support multimodal bioelectronic function.

## 8 Impact of Synthesis Temperature on Bioelectronic Materials Integration

Synthesis temperature plays a decisive role in determining whether disordered nanostructures can be integrated onto flexible, soft or bioresorbable substrates. This represents an essential requirement for next‐generation neural and glial interfaces. High‐temperature fabrication routes, including VLS growth of silicon nanowires or thermal oxidation of metal oxides, produce well‐defined crystalline phases but are fundamentally incompatible with polymeric supports, which typically degrade or deform above 150–250°C, unless using high glass transition temperature materials like polyimide (HGTTP), where in any case the direct growth of nanostructures is still challenging. In contrast, low‐temperature techniques such as hydrothermal ZnO growth (<95°C), LDH coprecipitation or electrodeposition at room temperature and plasma‐ or ion‐driven etching for nanograss Si enable the formation of complex, high‐aspect‐ratio nanostructures under conditions fully compatible with PET, PI, PDMS or elastomeric substrates (see Table 3). This thermal compatibility is crucial for neurointerfaces, where the mechanical compliance and conformability of the underlying substrate dramatically influence chronic stability and tissue response. Furthermore, low‐temperature fabrication pathways tend to preserve organic functional layers, adhesion promoters, or conductive polymer coatings that are often required to tailor impedance, interfacial chemistry or mechanical softness. Thus, the synthesis temperature not only dictates the feasible substrate ecosystem but also shapes the final bioelectronic properties of the interface, such as its softness, electrochemical behaviour and ability to sustain long‐term, inflammation‐free integration with neural tissue.

**TABLE 3:** Principal growth/deposition techniques to obtain disordered nanostructures.

Material	Technique	T (°C)	Compatible with flexible substrates	Notes
Si NW	MACE	20–60	Yes	Needs Si bulk
Si NW	PECVD growth	250–450	Only HGTTP	Nanostructures on PI
Si NW	RIE top‐down	<100		Nanograss
ZnO NR	Hydrothermal	70–95	Yes	Structures are etched by strong acids or bases
ZnO NR	Sol‐gel	150–300	Only HGTTP	PI is the best choice
ZnO NR	PLD	RT‐600	Only in the case of low T	
LDH NP/platelets	Coprecipitation	RT‐80	Yes	Good on PET, PI, PDMS
LDH NP/platelets	Electrodeposition	RT	Yes	It is used as a conductive substrate
Disordered films	GLAD	RT‐200	Yes	Fractal morphology

## 9 Impedance Mechanisms in Disordered Nanostructures

The presence of these peculiar nanotopographies are essential not only for the proper differentiation and growth of neural cells, but also to allow a specific interconnection with the surface of the electrode that can be surrounded, engulfed, or even partially internalized by the cells: this in turn results in different types of electrophysiological monitoring that spans from pure extracellular activity to full intracellular recordings [47, 72]. In case of pure extracellular recordings, the presence of nanopores, nanocavities and voids allows the formation of ionic micro‐reservoirs with a higher electrolyte exchange and higher chemical stability in terms of local pH and available charges. This provides a lower impedance between the cell membrane and the electrode surface with a higher signal‐to‐noise ratio with respect to planar electrode surfaces. Interestingly, these reduced impedance values are obtained even without the usage of conductive polymers like PEDOT:PSS or blends of graphene/graphene oxide nanoflakes and CPs [73], pushing at a new limit the electrical performance of the metal‐covered nanostructured electrodes (see Table 4).

**TABLE 4:** Estimated Areal Impedance for the Different Disordered Nanostructures.

Material	Morphology	Height	Base diameter	Z·A (Ω·cm^2^)	Dominant mechanism	Refs.
SiNWs (conical)	Disordered nanograss	1–2 µm	60–100 nm (tapered)	0.01–0.05	Double‐layer capacitance + seal resistance	[58]
SiNWs (cylindrical)	Dense vertical NWs	∼1 µm	∼100 nm	0.03–0.1	Capacitive, reduced sealing vs conical	[74]
ZnO nanorods	Disordered nanorods	0.8–1.5 µm	50–80 nm	0.05–0.2	Mixed capacitive/faradaic	[54]
Ni‐Fe LDH	Spherical‐like nanosheets	1‐2 µm	0.5–0.2 nm	14–18	Pseudocapacitive	[75]
Gold micro/nanostructured	Mushroom/ porous	1‐2 µm	µm‐scale	0.01	Purely capacitive	[76]

To enable a meaningful comparison between planar and disordered nanostructured electrodes, impedance values are commonly reported as areal impedance (Z·A), normalized to the geometric footprint. While this normalization does not capture the full increase in electrochemically active surface area induced by disorder, it provides a conservative metric that highlights how disordered nanostructures achieve one to two orders of magnitude impedance reduction without increasing the electrode footprint. The electrolyte used for impedance characterization was also carefully selected to minimize discrepancies and enable meaningful comparisons across different nanostructured interfaces. While cylindrical silicon nanowires and mushroom‐like structures (even at µm scale) provide low areal impedance values suitable for neural recording electrodes, layered double hydroxide (LDH) nanosheets primarily act as ionically active, pseudocapacitive coatings. Their higher areal impedance reflects a different interfacial function, emphasizing ionic buffering and chemical modulation rather than pure electronic charge transfer.

Additionally, by modulating the specific nanotopography of the disordered nanostructure, it is possible to accommodate each single type of cell population, potentially decoupling the neural and glial response, opening new insights into the glial‐glial and glial‐neuron communication. In literature, it has been already demonstrated on single astrocyte culture the peculiarity of some nanostructures [77, 78] in favouring the adhesion, growth and differentiation of astrocytes, allowing recording tiny signals coming from their basal activity or under chemical stimulation. SiNWs have also been implemented on neural co‐cultures, showing partial internalization in DRG cells [7]. These examples provide a new insight towards the realization of smart nanointerfaces enabling the recording and the discrimination of the physiological bioactivity of the different cell populations. Indeed, neurons generally are more attracted by the suitable nanodistributions of structures, while glial cells respond better to a mechanical and chemical uniformity of the underlying surface.

Finally, the presence of disordered nanostructured electrodes can allow a lower gliotic response, even without the use of drugs or chemicals, providing an environment potentially more similar to in vivo models, opening new avenues towards the implementation of more reliable in vitro models in accordance with the 3R principle. Indeed, these nanomaterials can foster the emergence of a new class of smart interfaces, enabling the development of increasingly complex organ‐on‐chip platforms to mimic and interrogate the functions and dysfunctions of neural and, more broadly, biological systems, thereby providing physiologically relevant insights that can reduce reliance on animal models while generating hypotheses and quantitative benchmarks to be subsequently validated in vivo.

## 10 Functional Readouts From Nanostructured Electrodes

Beyond morphological and electrochemical considerations, the most compelling validation of functional disorder in neural interfaces lies in its direct impact on electrophysiological readouts. A growing body of evidence demonstrates that disordered nanostructured electrodes consistently outperform flat counterparts in terms of signal‐to‐noise ratio (SNR), spike detectability, and channel activity, particularly in advanced in vitro models. This improvement does not arise from artificial hyperexcitability, but rather from enhanced neuron‐electrode coupling driven by increased effective surface area, reduced interfacial gap, and improved local seal resistance [6, 7]. For instance, nanostructured silicon‐based electrodes, including nanowire and nanograss architectures, have been shown to significantly reduce baseline noise and increase extracellular spike amplitudes compared to planar electrodes, enabling reliable detection of neuronal activity where flat electrodes often fail or exhibit noise levels exceeding 20 µV [58, 79].

**FIGURE 6:**
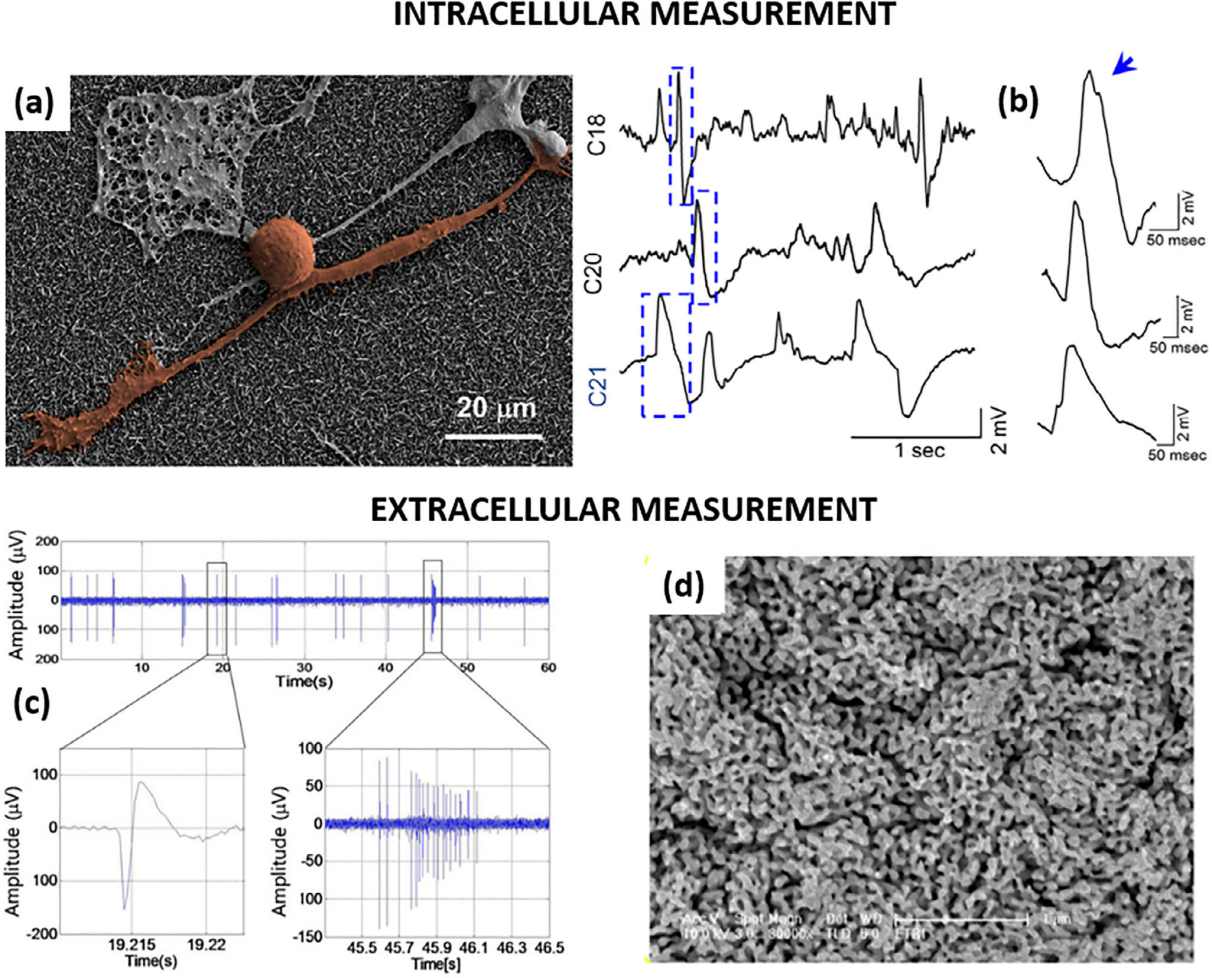
(a) SEM image of the astrocyte/DRG cell culture on Au/SiNW substrate (b) representative individual spikes randomly extracted from three different channels after adding capsaicin as stimulation factor (a and b courtesy of [7]); Adapted with permission [7]. Copyright 2025, John Wiley and Sons. (c) typical spontaneous signals from rat cortical neurons (recorded at DIV 19) for nanoporous Au‐modified electrode with magnified spike (bottom left) and burst (bottom right); (d) SEM image of the nanoporous Au‐modified electrode (c and d courtesy of [82]). Adapted with permission [82]. Copyright 2015, IOP Publishing.

In addition, robust neurons and glia differentiation on disordered spiky nanostructures are due to multiple factors derived from the local surface potential, the specific derived stiffness of the material, cell growth kinetics, etc. These features can promote neuron maturation with significantly lower times (e.g. 20 days) [80] with respect to similar spiky nanostructures but put in an ordered configuration [81] where neuron differentiation is achieved after 65 days. Moreover, vertically aligned nanowires result in a fixed configuration where the typical type of engulfment with neurons cannot be controlled. In case of disordered nanostructures, by changing the height of the wires and their growth tilted angle, it is possible to tune the orientation of the nanostructures, thus favouring a pure extracellular recording or a stronger contact with the cell, forcing a partial internalization of the wires [7]. A typical example of these two types of signals is reported in Figure 6, where primary Dorsal root ganglion (DRG) neurons co‐cultured with astrocytes and other glial cells are interfaced using gold‐coated silicon nanowire forest electrodes. In these cases, it has been demonstrated that the possibility of recording intracellular neuron activity with and without chemical stimulation through the partial internalization of the nanowires into the cell membrane. This distributed patch‐clamp‐like configuration enables intracellular access without extensive membrane rupture, thereby minimizing cellular damage and preserving membrane integrity. As a result, the approach supports stable and prolonged electrophysiological recordings in advanced in vitro models. On the other side, enhanced extracellular signals can be collected from nanoporous gold electrodes, adopting a disordered spongy mesh structure that facilitates the exchanges of ions and the local stability of the solution pH. In general, disordered nanoprotrusions and nanocavities can allow both extracellular and intracellular recordings by reducing the risks related to conventional patch‐clamp measurements and providing specific cell‐membrane junctions.

In particular, this is obtained through an intimate and localized cell‐electrode junction. As described by Guo [83], the tight nanojunction formed between the protrusion and the cell membrane creates a high‐resistance seal that amplifies extracellular signals, which are proportional to the temporal derivative of the transmembrane potential. Upon controlled membrane poration at the nanojunction, a resistive pathway is established between the cytosol and the electrode, allowing the recorded signal to contain a dominant fraction of the intracellular action potential superimposed with a smaller extracellular field contribution. Importantly, when such nanojunctions are distributed across a disordered nanostructured interface, functional disorder emerges as a key enabler of heterogeneous coupling regimes. While most nanoprotrusions operate in a predominantly capacitive, extracellular mode, a statistical subset establishes partial intracellular access through localized membrane poration. This spatially distributed and non‐uniform electrical coupling allows simultaneous extracellular and intracellular‐like recordings without requiring deterministic single‐site penetration, thereby enhancing signal robustness, reversibility, and long‐term viability. Functional disorder thus transforms local variability in nanojunction impedance into a global advantage for stable and high‐fidelity electrophysiological recordings.

## 11 Conclusions

This review proposes functional disorder not as a mere synonym of fractality, but as a unifying design principle based on non‐periodic multiscale heterogeneity with measurable interfacial consequences. Across the examples discussed here, disordered inorganic nanostructures simultaneously modulate adhesion, integrin‐mediated mechanotransduction, effective stiffness, porosity‐driven ionic exchange and enhanced cell‐electrode coupling, offering the possibility to obtain advanced neural interfaces. Across material classes, ranging from silicon nanowire forests to metal‐oxide nanorods and layered double hydroxide nanoplatelets, stochastic architectures consistently outperform planar or periodic counterparts in promoting superior cell adhesion and differentiation, reduced glial overactivation, and robust signal quality. Importantly, such functional disorder can be deliberately engineered using scalable, low‐temperature fabrication strategies compatible with flexible substrates and multimodal bioelectronic platforms. Looking forward, the integration of disordered nanointerfaces into advanced in vitro models, including organ‐on‐chip and hybrid neuroelectronic systems, holds significant promise for reducing reliance on animal models while generating mechanistic insights and quantitative benchmarks that can be selectively validated in vivo. Functional disorder thus emerges not as a limitation of fabrication, but as a powerful route toward biologically realistic and predictive neural interfaces.

## Conflicts of Interest

The authors declare no conflicts of interest.

## Data Availability

The authors have nothing to report.
